# Fabrication and evaluation of herbal beads to slow cell ageing

**DOI:** 10.3389/fbioe.2022.1025405

**Published:** 2022-12-08

**Authors:** Archna Dhasmana, Sumira Malik, Amit Kumar Sharma, Anuj Ranjan, Abhishek Chauhan, Steve Harakeh, Rajaa M. Al-Raddadi, Majed N. Almashjary, Waleed Mohammed S. Bawazir, Shafiul Haque

**Affiliations:** ^1^ Himalayan School of Biosciences, Swami Rama Himalayan University, Jolly Grant, Dehradun, Uttarakhand, India; ^2^ Amity Institute of Biotechnology, Amity University Jharkhand, Ranchi, Jharkhand, India; ^3^ Department of Biotechnology, Dr KNMIPER, Modinagar, Uttar Pradesh, India; ^4^ Academy of Biology and Biotechnology, Southern Federal University, Rostov-on-Don, Russia; ^5^ Amity Institute of Environmental Toxicology, Safety and Management, Amity University, Noida, India; ^6^ King Fahd Medical Research Center, King Abdulaziz University, Jeddah, Saudi Arabia; ^7^ Department of Community Medicine, Faculty of Medicine, King Abdulaziz University, Jeddah, Saudi Arabia; ^8^ Department of Medical Laboratory Sciences, Faculty of Applied Medical Sciences, King Abdulaziz University, Jeddah, Saudi Arabia; ^9^ Hematology Research Unit, King Fahd Medical Research Center, King Abdulaziz University, Jeddah, Saudi Arabia; ^10^ Animal House Unit, King Fahd Medical Research Center, King Abdulaziz University, Jeddah, Saudi Arabia; ^11^ Medical Laboratory Technology Department, Faculty of Applied Medical Sciences, King Abdulaziz University, Jeddah, Saudi Arabia; ^12^ Research and Scientific Studies Unit, College of Nursing and Allied Health Sciences, Jazan University, Jazan, Saudi Arabia

**Keywords:** herbal, quercetin, drug, graft, biocompatibility, herbal extracts

## Abstract

Several therapies and cosmetics are available commercially to prevent or delay cell ageing, which manifests as premature cell death and skin dullness. Use of herbal products such as *Aloe vera*, curcumin, vitamin C-enriched natural antioxidant, and anti-inflammatory biomolecules are potential ways to prevent or delay ageing. Eggshell membrane (ESM) is also a rich source of collagen; glycosaminoglycans (GAGs) also play an essential role in healing and preventing ageing. It is important to use an extended therapeutic process to prolong the effectiveness of these products, despite the fact that they all have significant anti-ageing properties and the ability to regenerate healthy cells. Encapsulated herbal components are therefore designed to overcome the challenge of ensuring continued treatment over time to prolong the effects of a bioactive component after *in situ* administration. To study their synergistic effects on a cellular level, alginate, Aloe vera, and orange peel extract were encapsulated in bio-polymeric foaming beads and modified with eggshell membrane protein (ESMP) at various concentrations (1 gm, 2 gm, and 5 gm): (A-Av-OP, A-Av-OP-ESMP1, ESMP2, and ESMP3). Analysis of the structural and functional properties of foaming beads showed interconnected 3D porous structure, a surface-functionalized group for entrapment of ESMP, and a significant reduction in pore size (51–35 m) and porosity (80%–60%). By performing DPPH assays, HRBC stabilization assays, and antibacterial tests, the beads were assessed as a natural anti-ageing product with sustained release of molecules effective against inflammatory response, oxidative stress, and microbial contamination. MTT assays were conducted using *in vitro* cell cultures to demonstrate cytocompatibility (in mouse 3T3 fibroblast cells) and cytotoxicity (in human carcinoma HeLa cells). Our study demonstrates that bio-polymeric ESMP beads up to 2 g (A-Av-OP-ESMP2) are practical and feasible natural remedies for suspending defective cell pathways, preventing cell ageing, and promoting healthy cell growth, resulting in a viable and practical natural remedy or therapeutic system.

## Introduction

In the current scenario, biogerontology is a primary area of focus for researchers and scientists (Juengst et al., 2003; Chopra, 2015). The ageing process is a natural phenomenon in which either a single cell or a whole organism undergoes cellular senescence. Significant factors in modern life that may lead to premature ageing include stress, unhealthy food or habits, drugs, smoking, and pollution (Mackenzie and Rakel, 2006; Burokas et al., 2017). Clinically, unhealthy skin may present as acne, wrinkles, dryness, or infections, which are key signs of ageing; the skin is the outermost protective covering of our body and interacts directly with the oxidative environment and harmful radiation (Jemec and Na, 2002; Pillai et al., 2005). Thus, the internal and external organs must be cleansed to overcome these issues and maintain healthy, wrinkle-free, and young-looking skin (Rittié and Fisher, 2015). It has been reported that cell apoptosis due to the shortening of telomeres, harmful UV radiation, glycation, oxidative stress, and hormonal stress causes ageing (Harley et al., 1990; Martin et al., 1993; Cunliffe et al., 2004). In the cellular system, oxygen-derived free radicals are the key players in cell damage, mutation, and early ageing at the cellular and tissue levels. The accretion of endogenous oxygen radicals generated in cells results in oxidative modification of biomolecules such as lipids, proteins, and nucleic acids and has been associated with the ageing and death of all living things (Finkel and Holbrook, 2000).

Cosmetic and therapeutic products, such as anti-ageing creams, masks, sunscreen lotions, moisturizers, and several other anti-ageing medicines, have been associated with prolonged side effects and poor outcomes (Kammeyer and Luiten, 2015; Panich et al., 2016). Herbs or medicinal plants enriched with phytochemicals have potential anti-inflammatory, anti-oxidative, and antimicrobial roles, thereby delaying cellular damage induced by external factors or internal cellular machinery (Pokorný and Schmidt, 2001). Natural antioxidants, such as phenols and flavonoids, slow the endogenous oxidation of a substrate at minute amounts, either through non-enzymatic mechanisms involving uric acid, glutathione, bilirubin, thiols, albumin, and dietary factors or through enzymatic mechanisms involving superoxide dismutases, glutathione peroxidases (GSHPx), and catalase (Sreeramulu et al., 2013). Dietary components have anti-inflammatory and anti-oxidative activity and act through different mechanisms. Free radical scavengers neutralize free radicals directly, reduce peroxide concentrations, repair oxidized membranes, and quench the oxidative capacity of catalytically active iron to decrease the production of reactive oxygen species and lipid metabolism (Miere et al., 2019).

Anti-ageing products contain chemicals from natural products, such as α-hydroxy acids, salicylic acid, hyaluronic acid, and ascorbic acid, along with preservatives for long-term shelf-life (Ahmed et al., 2020). In addition to natural herbal sources, animal products, such as egg shell membrane (ESM), are a rich source of anti-ageing biomolecules, which have traditionally been used as healing and tissue-regenerating biomaterial. Natural components are preferable in producing skin-friendly products. Herein, we report fabrication of a 3D bio-polymeric matrix for the treatment of premature cell ageing. Bio-polymeric hydrogel beads with Aloe vera, alginate, and orange-peel extract entrapped with eggshell membrane protein (ESMP) were synthesized and evaluated *via* physiochemical characterization and *in vitro* testing for applicability as an anti-ageing product.

It was reported that the lubricant aspect of Aloe vera, enriched with vitamins and fibres, has antioxidant and hydrating gel properties that protect the skin from radiation and thermal or solar burn. It has a prophylactic effect on damaged skin tissue through natural hydration and an antibiotic effect that leads to soothing, cooling, increased elasticity, and promotion of the youthful properties of skin. Clinically, different products are approved to treat skin problems, promote wound healing, slow ageing, heal scratches, and as cleansers to purge the body or skin of impurities (Roth et al., 2001; Surjushe et al., 2008).

Another essential component of healthy skin is hyaluronan or hyaluronic acid. It is a significant component of ESM and has the potential to retain water molecules by efficiently binding to them, thereby maintaining the moisture content of the cell (Papakonstantinou et al., 2012; Burokas et al., 2017). Additionally, the organic components hyaluronic acid, proteoglycans, and collagen in ESM enhance cellular activity and collagen synthesis and inhibit the effects of UV light exposure, thereby preventing premature cell-ageing and inflammation and promoting regeneration of healthy cells (Zhao and Chi, 2009; Kulshreshtha et al., 2020). Hence, ESM is a potentially rich source of components from which to produce beauty products, such as anti-inflammatory creams/lotions, anti-wrinkle agents, antimicrobial wound-healing agents, and moisture-retaining products (Pinsky, 2017; Marimuthu et al., 2020).

Citrus fruits are rich sources of vitamin C, and α-hydroxy acid has natural antibacterial and antioxidant properties. They play vital roles in deferring ageing by enhancing the synthesis of collagen to improve the elasticity and flexibility of the dermal layer of the skin (Pullar et al., 2017; Miastkowska and Sikora, 2018). Products containing citrus fruit extracts have been used medically and sold commercially as skin lighting, dark-spot removal, and anti-wrinkle agents.

Researchers have fabricated alginate beads for the entrapment of bioactive or drug molecules and made polymeric blends containing *Aloe vera*, ESM hydrolysate, and curcumin; these natural matrices for the delivery of therapeutic products for wound dressing and tissue regeneration have demonstrated significant benefit (Pereira, 2013; Shi et al., 2014; Yagi et al., 2018).

**FIGURE 1:**
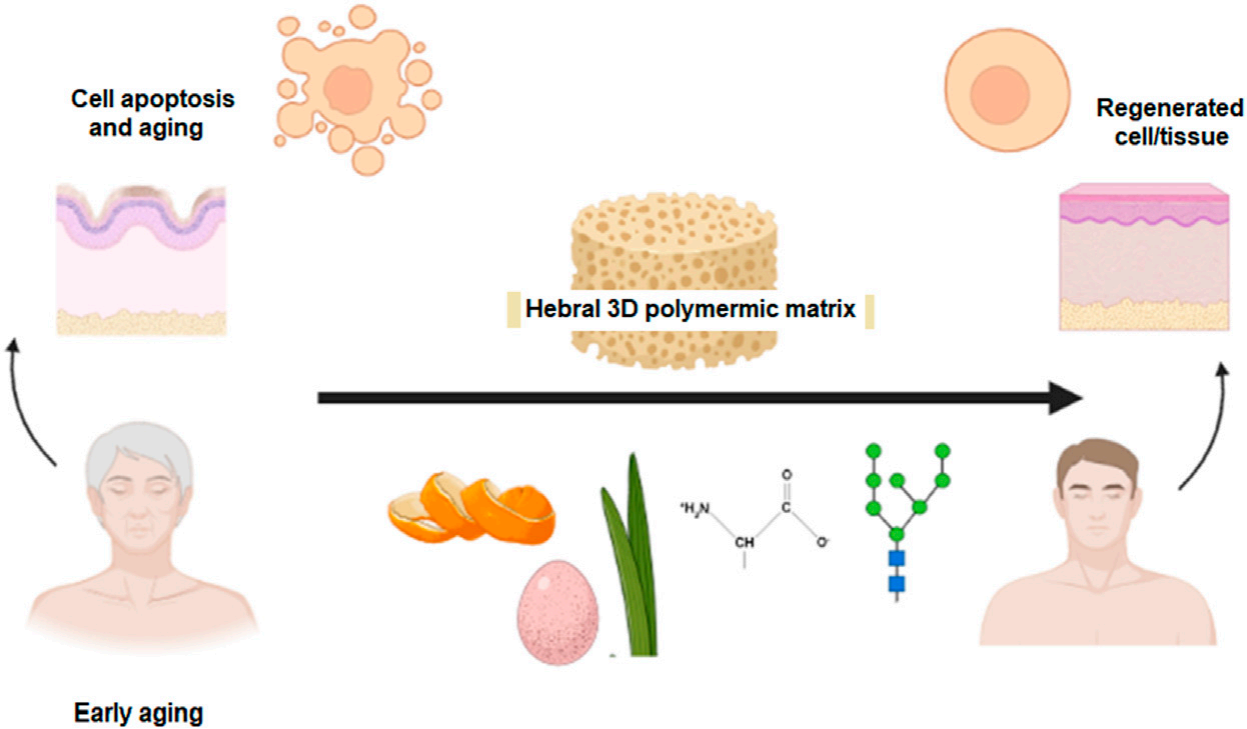
Graphical abstract.

The importance of this study is in the development of polymeric beads from natural sources and evaluation of the beads in their capacity to be used in a skin-friendly, biocompatible, non-allergenic, and biodegradable form that is eco-friendly. The bio-polymeric blend of alginate, orange peel powder, and *Aloe vera* enriched with bioactive agents was used in fabrication of the foaming beads with entrapped ESMP at different concentrations (Figure 1). The foaming beads were used as a natural 3D bioactive porous matrix that is a novel formulation for study of the synergistic effects of all components on cellular pathways related to tissue regeneration and remodeling, with the aim of delaying cell apoptosis and/or ageing.

## Materials and methods

### Materials

Raw material for fabricating bio-polymeric beads was collected, including raw eggshell, orange peel from the campus canteen, and fresh *Aloe vera* leaves from the kitchen garden. Other reagents, including alginate, ethanol, phosphate-buffered saline (PBS), calcium chloride, and acetic acid were purchased from HI media Laboratories, India. All the reagents/chemicals used for fabricating hydrogel beads and in their characterization were of cell culture grade.

### Bead fabrication

Bio-polymeric herbal beads of alginate, Aloe vera, and orange peel extract with entrapped ESMP were synthesized using the drop-extrusion crosslinking method in calcium chloride solution. Subsequently, characterization of fabricated samples was carried out to evaluate their applicability in anti-ageing and cell regeneration processes.

(a) ESMP preparation: ESMP solution was prepared according to the previously published protocol (Strohbehn et al., 2013), which is described as follows: briefly, the collected raw eggshell was thoroughly cleaned with double distilled water (ddH2O) and dried at 50°C for 30 min. After drying, the eggshell was soaked in 70% acetic acid for 24 h, with subsequent washing of the decalcified eggshell membrane with ddH2O. Next, the ESMP was dried at 50°C for 4 h and a fine powder was obtained by crushing the ESMP in liquid nitrogen. The ESMP powder was dissolved and incubated in an alkaline solution of sodium hydroxide (NaOH) overnight at 50°C. The dissolved sample was centrifuged and the supernatant collected. ESMP from the solution was then neutralized and lyophilized to form a powdered sample that was stored in the refrigerator.
(b) Preparation of orange peel powder: Collected orange peel was thoroughly washed in ddH2O and dried in a hot-air oven at 37°C. After drying, a fine powder was prepared using a food grinder. The powder was stored in air-tight plastic tubes.
(c) Extraction of Aloe vera gel: Fresh Aloe vera leaves were collected from the college herbal garden. The leaves were washed thoroughly with ddH2O, and the top and bottom layers of the leaves were peeled off. The thick, clear gel, or pulpy content of the leaves was collected at the centre. Subsequently, the pulp was blended in a grinder to produce a clear Aloe vera extract.
(d) Preparation of gel beads: Bio-polymeric beads were prepared following the previously published protocol, with some modification in bead composition (Sharma et al., 2012). We generated stable foam containing the bio-polymeric solution with alginate (3 wt %) and orange peel powder (2 wt %) in Aloe vera juice (w/v), which was incubated at 40°C for 2 h on a magnetic stirrer. After mixing, the polymer beads were prepared *via* simple drop-extrusion methods (21G syringe at a height of 10 cm), using a solution of CaCl_2_ (1 wt %) as a crosslinking agent and ESMP at different ratios, as indicated in Table 1. ESMP was included at different concentrations (1 gm to 3 gm in 100 ml solution) to study physiochemical interaction, dose-dependent effects, and outcome variability in biological effects, as discussed in the following sections.

**TABLE 1:** Composition of the components used to fabricate the ESMP-loaded alginate (A), *Aloe vera* (Av), and orange peel (OP) extract bio-polymeric beads.

Sample type	Bead composition				
	Alginate (%)	Orange powder (%)	*Aloe vera* (ml)	ESMP	CaCl_2_ (%)
Control (A-Av-OP)	3	2	100	−	1
Sample 1 (A-Av-OP-ESMP1)	3	2	100	1 gm	1
Sample 2 (A-Av-OP-ESMP2)	3	2	100	3 gm	1
Sample 3 (A-Av-OP-ESMP5)	3	2	100	5 gm	1

Fabricated beads were kept in crosslinking solution for 5 min to facilitate efficient crosslinking and ESMP entrapment, then gently washed with ddH2O to remove unbound particles and excess CaCl_2_. Lastly, the beads were filtered and separated from the solution, then freeze-dried for prolonged storage.

### Morphological analysis

The macro-structure of the bio-polymeric beads was determined visibly by macroscopic examination and size dimension (diameter in mm) measurement of a randomly selected 50 beads from each sample using a vernier caliper. However, ultra-structure of the beads was analyzed by studying the cross-sectional area of images of five randomly collected beads captured using a field emission scanning electron microscope (FESEM; QUANTA 200F FEI, Netherlands). The pore size and interconnectivity of the beads were determined by analyzing microscopic images using ImageJ software, and the average dimensions of the beads were determined.

### FTIR analysis

The functional and intermolecular interaction that occurs between the samples was determined by FTIR spectra analysis. All the samples were prepared following a previously published protocol (Mackenzie and Rakel, 2006). Briefly, each sample bead was mixed with KBr at a ratio of 1:900 and ground to a fine powder for FTIR analysis. The absorbance of each sample at wavelength 4000 to 400 cm^−1^ at 2 cm^−1^ resolution was assessed using an FTIR spectrophotometer (Thermo Nicolet, United States), and the generated spectra were analyzed.

### Porosity and swelling

Good porosity is required for absorption, vascularization, transport, and release of drug molecules. Therefore, we measured the porosity (χ) of the beads using the liquid dispersion method (Sharma et al., 2012). We measured the void space in the 3D interconnected polymeric beads available to be filled up with the components of the drug/biomolecule by analyzing SEM images of the beads using Image J software, as mentioned previously. The liquid dispersion method and the following equations were used to measure the porosity and the full volume of the freshly prepared foam bead sample (*n* = 5):

$$\chi =\frac{V_{1}-V_{3}}{V_{t}}\times 100,$$ (1)

$$V_{t}=V_{2}{-V}_{3}.$$ (2)

In brief, polymeric beads were transferred to a graduated cylinder with a known volume of solvent (V_1_); then the volume of solvent after immersion of the beads, V_2_, was measured. The volume, V_3_, was also measured after removal of the beads at regular time intervals. V_t_ represents the total volume.

Similarly, bead hydrophilicity, or swelling ratio (S), was determined by immersing the beads in 1X PBS solution for 1 h at room temperature (RT) and measuring their weight at a regular time interval of 10 min. The following equation is used to measure S (Eq. 3). Here, W_s_ is the weight of wet beads and W_d_ is the weight of dried beads.

$$S=\frac{W_{s}-W_{d}}{W_{d}}\times 100.$$ (3)

All the experiments were repeated five times (*n* = 5), and the mean value was calculated.

### *In vitro* degradation and drug release profile

The rate of degradation of beads was used to measure their stability and was determined under non-enzymatic conditions in a PBS buffer system (Dhasmana et al., 2019). Briefly, 5 gm of each sample bead was incubated in 10 ml 1X PBS (pH 7.4) at 37°C. Beads were taken out of the buffer at regular intervals for weight measurement. The biodegradation rate or weight loss (*n* = 3) was calculated using Eq. 4:

$$weight\;loss\;\%=\frac{W_{o}-W_{t}}{W_{o}}\times 100,$$ (4)

where W_0_ denotes the initial weight of the scaffold and W_t_ denotes the weight of the degraded scaffold at different time intervals.

The release of bioactive components from the polymeric bead surface after incubation in PBS buffer was measured using the total immersion method. The absorbance of the ESMP encapsulated bead samples was recorded using a UV-Vis spectrophotometer, and the calibration curve of ESMP (50 μg/ml) was prepared for monitoring drug release. The modified beads were immersed in 150 ml of 1X PBS and placed in a shaking water bath at 37°C. After 12 h of incubation, 2 ml of the sample were taken out at regular time intervals of 30 min, and the absorbance at 412 nm was recorded. After measuring absorbance, the sample was reintroduced into the main flask to maintain constant final volume. The drug release percentage was obtained by comparison with the standard calibration curve.

### Antibacterial activity

*In vitro* antibacterial activity of the beads was tested against Gram-positive (*S. aureus*) and Gram-negative (*E. coli*) bacteria, as described previously (Dhasmana et al., 2019). Briefly, nutrient media was prepared. Subsequently, 2 ml of bacterial suspension or aliquots were added to a culture tube containing 10 ml MH broth, which was then labelled as the positive control culture. However, for the test sample, 5 mg beads of each sample (control and ESMP beads) were added to the culture tubes, along with MH broth and bacterial culture. After inoculation, all the culture tubes were placed in a shaker incubator at 37°C. Absorbance of the culture samples at 570 nm at different predetermined time intervals were measured by taking 2 ml of the liquid culture medium from each sample group. The generated data were then plotted as a bacterial growth curve through which the antibacterial effects of the fabricated sample beads were assessed.

### Anti-oxidation analysis

Antioxidant activity of the fabricated polymeric beads was measured *in vitro* using the DPPH assay, as previously described (Eq. 5) (Enkhtuya et al., 2014). Briefly, 0.2 mM DPPH solution in 200 µL absolute ethanol and 800 µL 0.1 M Tris–HCl buffer (pH 7.4) was prepared and kept in the dark at RT. Reagents were then sequentially added to test tubes as follows: Blank contained 3.3 ml ethanol and 0.5 ml sample; the negative control contained 3.5 ml ethanol and 0.3 ml DPPH solution; and the test sample contained 0.5 ml sample, 3 ml absolute ethanol, and 0.3 ml DPPH solution. All samples were mixed well, followed by incubation for 30 min at RT. The absorbance (Abs) of each sample at 517 nm was measured using a UV-vis spectrophotometer (DU 800; Beckman Coulter, Fullerton, CA, United States).

$$AA\%=100-\left[\frac{Abs_{sample}-Abs_{blank}}{Abs_{control}}\right]\times 100.$$ (5)

### Anti-inflammatory activity

For estimation of the anti-inflammatory activity of the herbal beads, the HRBC membrane sterilization method was used, as previously described (Chowdhury et al., 2014). Briefly, blood samples were collected from a healthy donor and mixed with an equal volume of sterilized Alsever’s solution. Next, the blood was centrifuged at 3000 rpm to separate erythrocytes (RBC), which were then washed thoroughly with saline solution (three times), and resuspended at 10% v/v in saline solution.

Different concentrations of sample extract were then mixed separately in 1 ml PBS buffer, 2 ml hyposaline, and 0.5 ml HRBC suspension and incubated at 37°C for 30 min. Following incubation, samples were centrifuged at 3000 rpm for 20 min, and the absorbance of the supernatant at 560 nm was calculated to estimate haemoglobin content and the hemolysis percentage. Here, we set the negative control group as 100%, thus allowing calculation of HRBC membrane stabilization or protection percentage using the following equation.

$$HRBC\;membrane\;protection=100-\left(\frac{Abs\;of\;test\;sample}{\;absorbance\;of\;control}\right)\;100.$$ (6)

### *In vitro* biocompatibility

*In vitro* cell culture was used to assess biocompatibility and measure the effects of polymeric herbal bead components on cell viability and proliferation (Dhasmana et al., 2019). Briefly, 10 µl cell suspension (∼1×10^3^ cells), of either 3T3 mouse fibroblast cells or HeLa carcinoma cells, were seeded over the beads (control: unmodified beads; Samples 1, 2, and 3: ESMP-modified beads at different concentrations) in the tissue culture plate (TCP) wells. TCP wells containing cells cultured without beads were the negative controls. Subsequently, fresh DMEM nutrient medium (990 µl) was added to each TCP well and cells were placed in a CO_2_ incubator at 37°C. After 24 h incubation, cell growth and viability were assessed using the MTT assay and by measuring the absorbance of the samples at a wavelength of 490 nm at 1, 3, 5, and 7 days using a microplate reader (TECAN, India).

### Statistical analysis

All the experimental data and results were measured quantitatively and are indicated as mean ± standard deviation, with statistical analysis *via* analysis of variance (ANOVA) and calculation of p-value (<0.05) as an indicator of statistical significance.

## Results and discussion

### Morphological analysis

**FIGURE 2:**
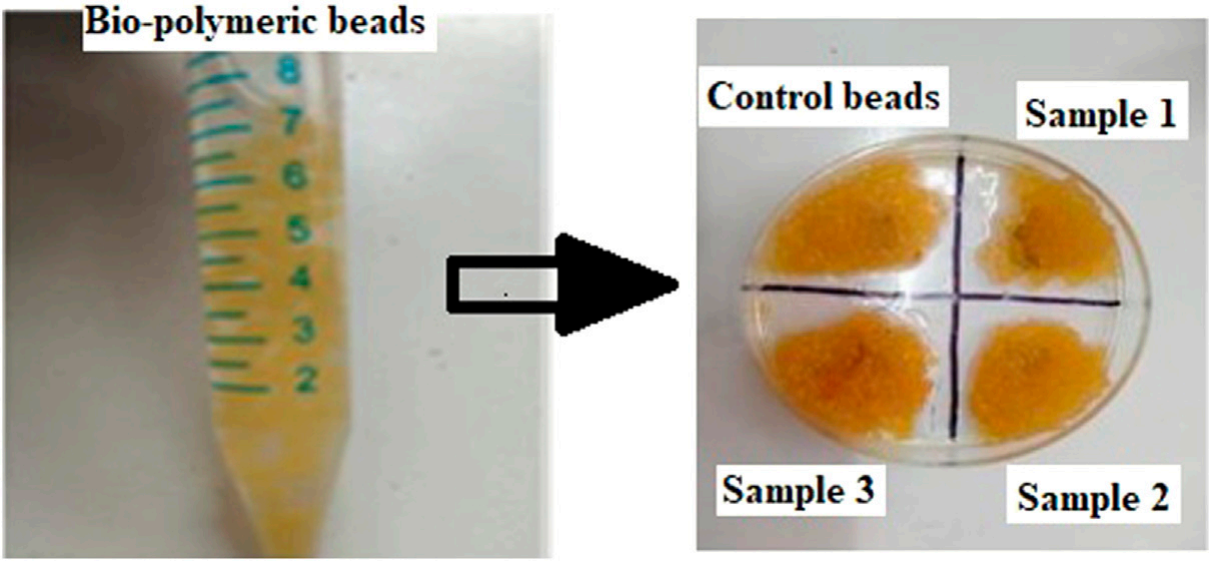
Images of the prepared bio-polymeric alginate, *Aloe vera*, and orange peel extract beads containing different concentrations of ESMP.

**FIGURE 3:**
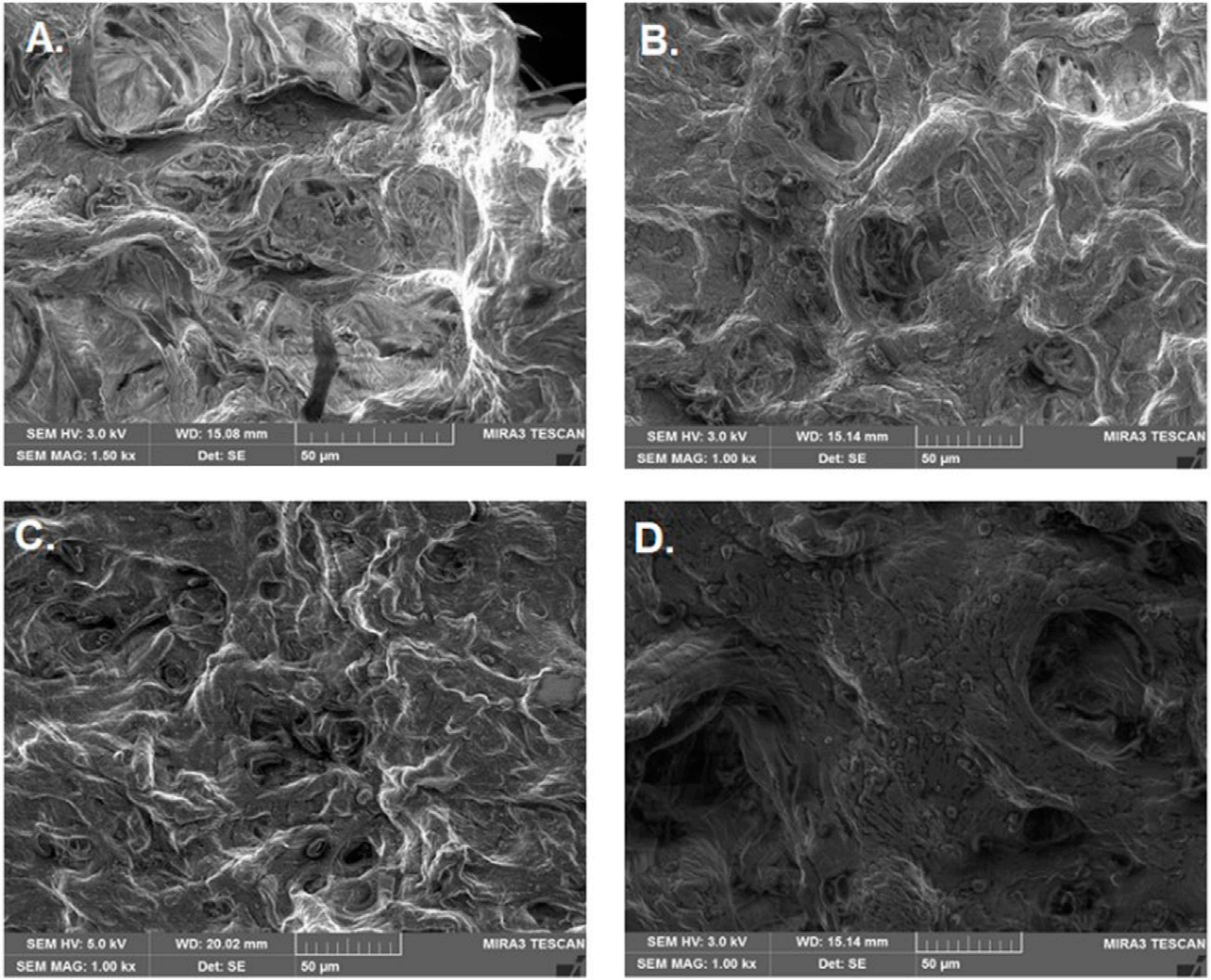
Ultrastructure of bio-polymeric beads. **(A)** Control (alginate, *Aloe vera*, and orange peel powder) and **(B–D)** modified beads having different concentrations of ESMP (1, 3, or 5 mg).

Bead morphology and relative size measurements (mean ± SE) were conducted through simple visualization using microscopy. The bead shape was spherical, and bead size ranged from 0.5 to 0.8 mm in diameter (Figure 2; Table 2). The bead ultrastructure represented by the cross-sectional area showed good porosity and pore size ranging from 35 to 50 µm (Figure 3). Good porosity and pore size provide better diffusion of nutrients, vascularization, and migration of cells inside the beads (Tayla et al., 2011). In a previous study, natural polymeric foaming scaffolds had good porosity of up to 90%, with spongy 3D ultrastructure for tissue engineering applications (Sharma et al., 2015). Here, the viscous and unamphiphilic nature of alginate provided good foam stability, and orange peel particles provided stability to the crosslinked matrix. Additionally, the crosslinking of the polymeric beads was performed without using any toxic crosslinking agent, but rather simple alginate bead ionic crosslinking with calcium chloride. The porous matrix was designed for the enhanced encapsulation and release of bioactive components, cellular adherence, growth, and proliferation.

**TABLE 2:** Macroscopic size (mm) and pore size (µm) dimensions of fabricated bio-polymeric beads.

Sample type	Bead size (mm)	Pore size (µm)
Control	0.5 ± 0.02	51.01 ± 0.04
Sample 1	0.56 ± 0.10	42.2 ± 0.082
Sample 2	0.73 ± 0.08	40.15 ± 0.05
Sample 3	0.82 ± 0.05	35.22 ± 0.02

### Porosity and swelling ratio

**FIGURE 4:**
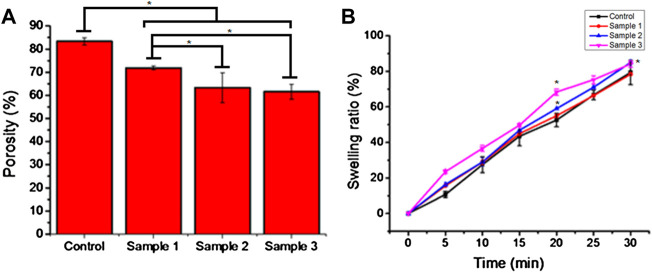
Porosity **(A)** and swelling ratio **(B)** of the bio-polymeric beads in 1X PBS solution indicate the hydrophobicity and voids in the control and ESMP-modified beads (samples 1, 2, and 3). Here, * indicates the significant difference between samples (*p* < 0.05).

The porosity of beads indicates the diffusion rate of molecules through the cell membrane. All the beads demonstrated good porosity, and the porosity of the control sample beads was 80%. However, the porosity of the ESMP-containing beads was significantly reduced as we increased the concentration of ESMP, i.e., 80%–60%, which demonstrates the significant difference in the adsorption of the ESMP particles on the surface of the polymeric matrix (Figure 4A).

The bead swelling ratio or absorption rate showed an excellent rate of sorption and moisture retaining capability of the bio-polymeric beads, i.e., up to 80% (Figure 4B). In a previous study, hydrogel fabricated from alginate and *Aloe vera* pulp with good water absorption capacity resulted in the removal of exudate from the wound for better healing (Pereira, 2013).

The chemical composition of bio-polymeric beads has a significant amount of polymeric sugar and amino derivatives along with hydrophilic compounds present in the base-solvent, i.e., Aloe vera pulp, resulting in an excellent swelling ratio and hydrophilicity (Ranjbar-Mohammadi, 2018). Among all the samples, ESMP-containing beads changed the sorption rate most significantly compared to the control sample due to the enrichment of proteins and peptides. Researchers have fabricated ESMP-containing nanofibrous scaffold for healing cutaneous wounds due to their moisture-retaining ability (Mohammadzadeh et al., 2019). In another study, researchers reported that the soluble ESMP proteins used to synthesize hybrid grafts resulted in a controlled sorption rate and strength for better cellular interaction (Choi et al., 2020). Kevin (2020)also reported that the bio-polymeric film containing orange peel powder improves matrix stability and retains moisture for a prolonged time. Thus, all the constituents play an essential role in maintaining the polymeric bead sorption or hydrophobicity for retention of moisture and healthy skin.

### FTIR analysis

**FIGURE 5:**
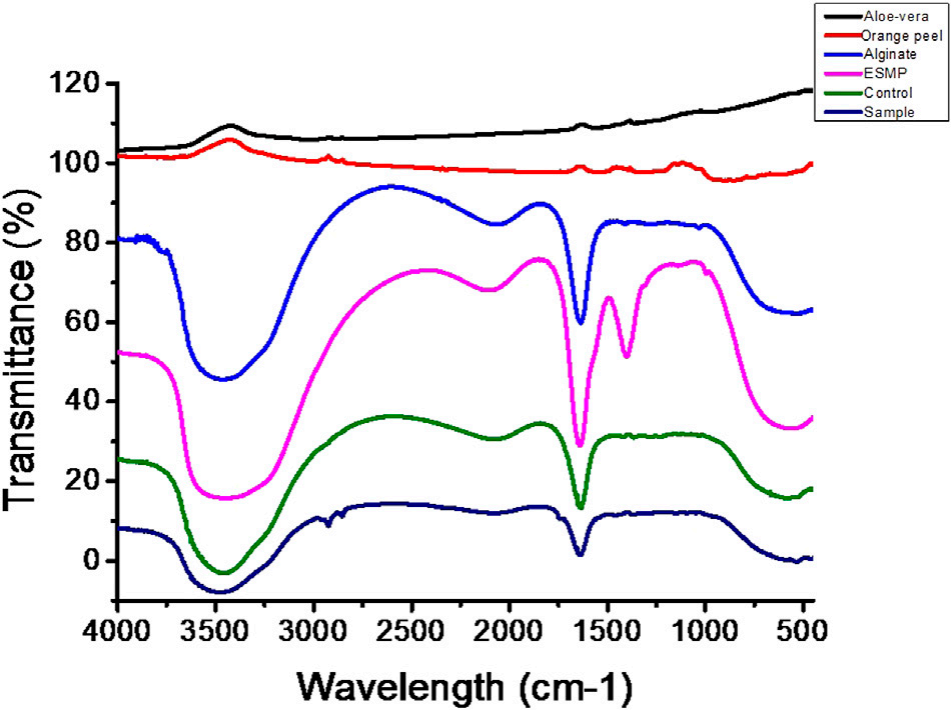
FTIR spectra of the bio-polymeric bead components, including those of control bio-polymeric beads (alginate, *Aloe vera*, orange peel), and ESMP-modified sample beads (alginate, *Aloe vera*, orange peel, ESMP).

The chemical interaction and linkage between the different constituents of bio-polymeric beads assessed *via* FTIR analysis suggested good interaction and bonding (Figure 5). The FTIR spectra of bio-polymeric bead components indicated pure extraction by the presence of functional peaks: orange peel extract—3289 cm^−1^ (O–H stretching), 1750 cm^−1^ (C=O stretching), 1267 cm^−1^ (amide III C–N group), and 1001 cm^−1^ (C–S); Alginate—3445 cm^−1^ (O–H vibration) and 1729 cm^−1^ (C=O stretching) for glucuronic and mannuronic acid units; Aloe vera—3438 cm^−1^ (O–H vibration), 2920 cm^−1^ (symmetric = CH_2_ stretching), 1721 cm^−1^ (C=O stretching), and 1580 cm^−1^ (amide II, N–H bending); and ESMP˗ 3492 cm^−1^ (O–H, N–H stretching), 2125 cm^−1^ (C–H stretching), 1635 cm^−1^ (amide I C=O stretching), 1416 cm^−1^ (N–H stretching), and 587 cm^−1^ (C–S stretching), respectively. In the case of control beads of alginate, orange peel, and Aloe vera, the shifting and disappearance of orange peel and Aloe vera functional peaks indicated bonding between the functional groups. However, the stretching mode formed peaks at 3487 and 1742 cm^−1^ for the C–O, O–H, and N–H groups, with asymmetric stretching vibration of C–H bonds. Correspondingly, the ESMP-modified bio-polymeric bead sample showed the same stretching and shifting peaks as observed in the control group. Stretching at 1635, 1416, and 587 cm^−1^ relates to the stretching modes of C-O, N-H, and C-S bonds. The FTIR data confirmed the complete linkage between the functional groups of bio-polymeric bead constituents and free functional groups for better cell attachment. Likewise, other scientists have fabricated biodegradable scaffold and reported peaks for all the essential proteinaceous compounds in the regions: 3200–3500 cm^−1^, 1600–1700 cm^−1^ (amide I), 1500–1600 cm^−1^ (amide II), and 1200–1300 cm^-1^ (amide III), respectively (Hosseini et al., 2017; Anchor and Zaghouane-Boudiaf, 2020). In another study, FTIR spectra of orange peel extract encapsulated in Ca-alginate beads indicated that the crosslinking between the functional groups results from long-term stability and biological activity of the encapsulated component (Savic Gajic et al., 2021).

### *In vitro* biodegradability and release profile

**FIGURE 6:**
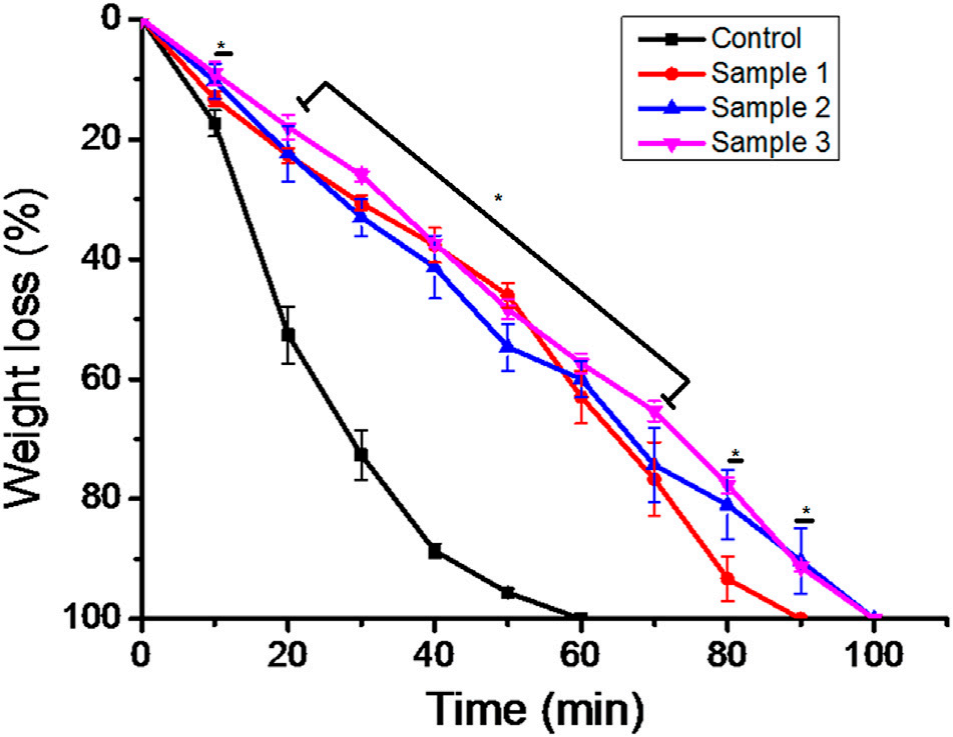
*In vitro* biodegradation of the bio-polymeric beads, including control and ESMP-modified samples, indicating weight loss in the non-enzymatic system of 1X PBS. Here, * indicates the significant difference between samples (*p* < 0.05).

The biodegradability of the ESMP-modified bio-polymeric beads varied as we increased the concentration of ESMP in the PBS solution (Figure 6). The weight of the sample beads decreased per data collected at regular time intervals and the beads degraded completely within 100 min. In contrast, the control beads degraded more rapidly, within 60 min of incubation, when compared to modified beads, which had prolonged time to degradation. The sample begins losing weight and, at a particular time, the level of biodegradability is constant for both samples 2 and 3. Sample 1 degraded quite early, within 90 min after the control sample, and both final samples degraded after 100 min at constant intervals. In a previous study, polymeric *Aloe vera* showed highly improved water sorption properties that resulted in a faster degradation rate due to the degradable cleavage of the polymeric network (Pereira, 2013). However, in another study, the ESM-*Aloe vera* nanofiber degradability rate significantly improved under prolonged incubation due to the leaching and hydrophilic nature of the ESM (Mohammadzadeh et al., 2019). Similarly, to improve the degradability of gellan gum, ESM was incorporated into the hydrogel fabrication, which then showed up to 30% faster degradation compared to the pure gellan gum hydrogel due to the hydrophilicity of ESM components (Choi et al., 2020). Thus, the complete degradation of all samples in non-enzymatic 1X PBS solution resulted in continuously decreasing weight after a specific time interval.

**FIGURE 7:**
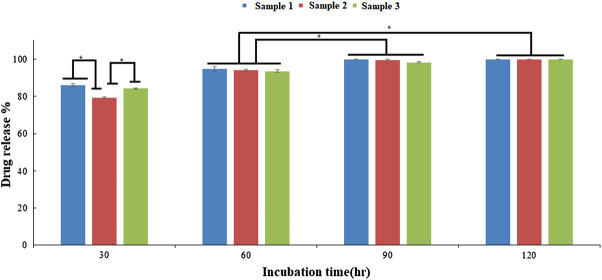
*In vitro* drug release profile of the ESMP entrapped bio-polymeric beads showed constant slow release of ESMP after 30 h incubation and significant progression in release over time (*p-value < 0.05).

Additionally, the bead degradation and the release of active components is mandatory for measurement of their effects *in situ*. The degradation of the scaffold or matrix after implantation *in situ* results in the formation of short peptides, molecules either having a toxic effect or activating a signalling response in the dividing cells. Therefore, measuring the biodegradability and release of active components from the matrix is necessary. In recent studies, the alginate-modified drug beads have released dosages for constant and extended-release drug products (Tønnesen and Karlsen, 2002; Liew et al., 2006; Ning et al., 2018). To enhance the bioactivity of the beads, ESMP modification was carried out, and their release profile was measured, showing an initial burst release of up to 80% and subsequent controlled release of the ESMP particles entrapped in the matrix over time (Figure 7). The further compatibility of the bead matrix after degradation and release of biomolecules was measured using *in vitro* cell culture.

### Antimicrobial activity

**FIGURE 8:**
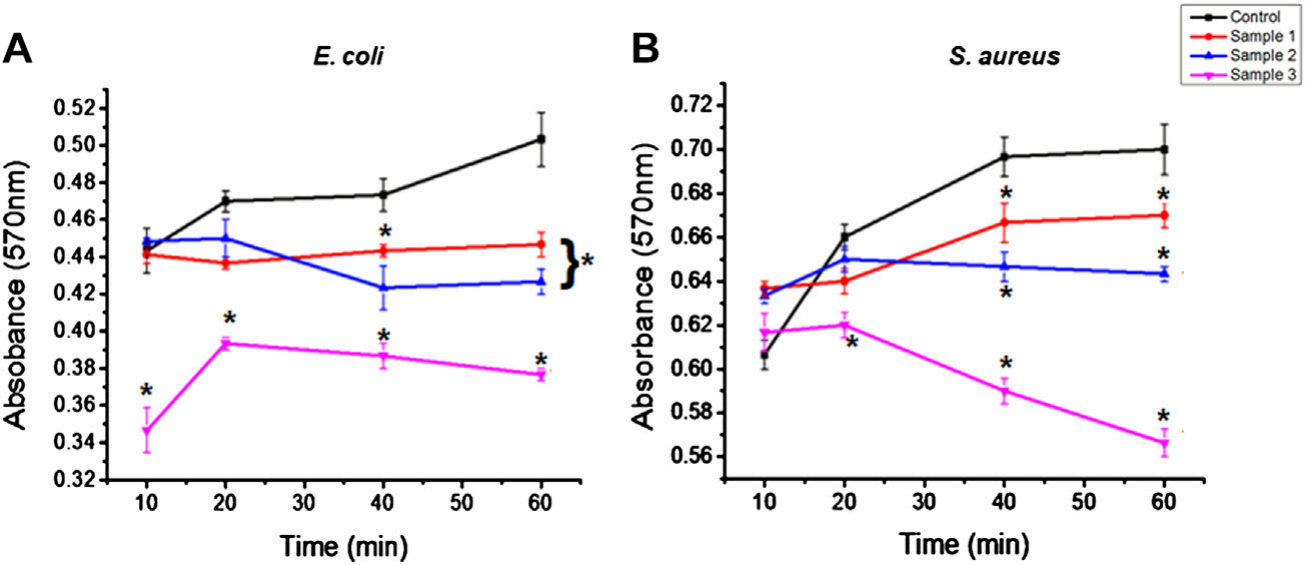
Antibacterial effects of the bio-polymeric beads, including control and modified samples, *via* inhibition of the growth of **(A)** Gram-positive and **(B)** Gram-negative bacteria. Here, * indicates the significant difference between samples (*p* < 0.05).

The antimicrobial activity of the bio-polymeric beads examined against both the Gram-positive *Escherichia coli* (*E. coli*) and Gram-negative *Staphylococcus aureus* (*S. auerus*) bacterial strains suppressed growth of bacterial cells over time (Figure 8). The natural component inhibited the growth of bacterial cells in the culture medium and was effective against both bacterial strains. In the control sample, bacterial cells grew steadily, but after 40 min there was a constant slope, as shown. Similarly, sample 1, after 20 min, showed the progressive growth of microbial cells, but at a lower rate than with the control beads. However, in samples 2 and 3, bacterial growth was suppressed, with a constant decrease in absorbance demonstrating the effective antibacterial properties of ESMP hydrolysates in the culture medium. With increased concentration of ESMP in the samples, the reduced absorbance indicated significant bacterial growth reduction and antibacterial effects. A previous study conveyed the antibacterial effects of the short bioactive peptide (e.g., cationic peptides and eicosanoids) at the wound site for better healing of infectious wounds (Zhang et al., 2000). It has been reported that the ESM hydrolysates have antimicrobial activity against several bacteria, including *E. coli* and *S. aureus*, suggesting their potential application to treat acne (Yoo et al., 2014).

Kevin (2020)showed that the biological property of the gelatin film improved along with increased antimicrobial effects after incorporating the orange peel powder against *E. coli* and *S. aureus*. Correspondingly, the bacterial growth in the control and modified sample beads was observed at a high rate, but gradually decreased with a time interval of 20–40 min. The antibacterial activity of ESMP was observed to be significantly better for *E. coli* than *S. aureus* and to gradually decrease during the incubation time. Thus, many researchers have fabricated skincare or healing agents containing particalized ESM for increased antimicrobial activity and superior anti-inflammatory activity against skin-associated pathogens (Li et al., 2019; Kulshreshtha et al., 2020).

### *In vitro* antioxidant and anti-inflammatory activity

**FIGURE 9:**
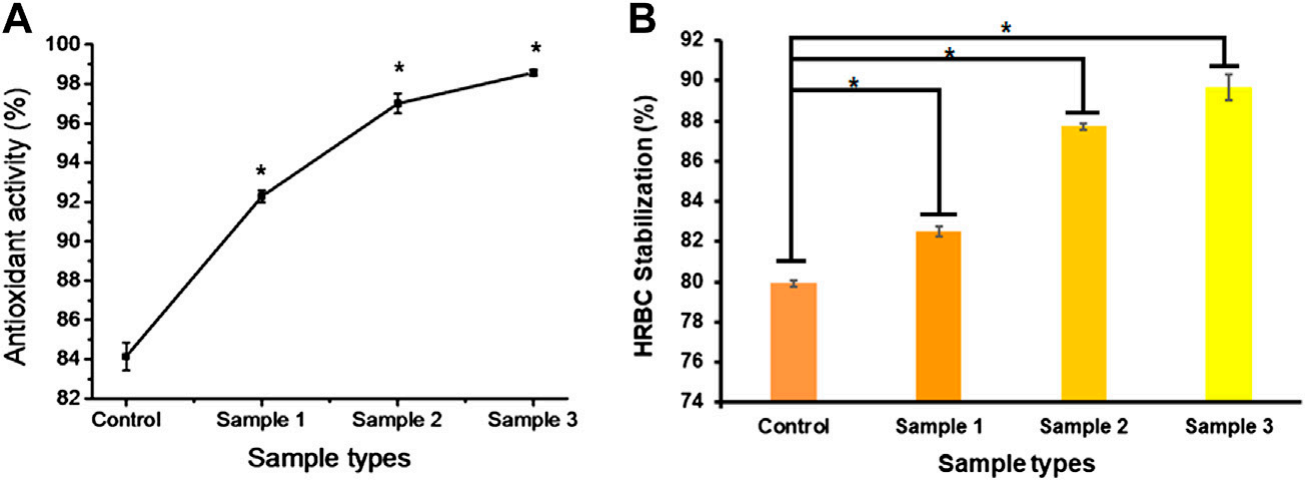
*In vitro* anti-oxidative **(A)** and anti-inflammatory **(B)** effects of bio-polymeric beads indicate significant antioxidant activity and cell lysis protection by natural components. Here, * indicates the significant difference between samples (*p* < 0.05).

Inflammation is required for better and faster healing of wounds, but prolonged inflammation and oxidative stress due to the formation of free radicals result in chronic wounds and mutations. Thus, anti-inflammatory and anti-oxidative agents are required to suppress the prolonged inflammation, swelling, and evoked immune system to protect the cell from free radicals and delay cell death. It was reported that standard dietary agents enriched with anti-oxidative molecules suppress or inhibit the reactive oxygen species and oxygen-derived free radicals that may result in cellular ageing (Fusco et al., 2007). Many researchers found that the natural components of *Aloe vera*, orange peel, and ESM possess antioxidant and anti-inflammatory properties (Yagi et al., 2002; Das et al., 2011; Yoo et al., 2014). In our study, the bio-polymeric beads showed up to 80% antioxidant activity, with constant increase after the incorporation of ESMP powder at higher concentration in sample 3, i.e., up to 98% (Figure 9A). The HRBC stabilization assay is a standard method to measure the anti-inflammatory activity of drug components or molecules based on the RBC lysis percentage, given the similar cell membrane composition of RBC and lysosomes.

All the bio-polymeric beads showed up to 90% HRBC stabilization. They showed constantly reduced anti-inflammatory effects with increased ESMP powder concentration, i.e., from 90%–80% (Figure 9B), indicating a significant anti-inflammatory effect on red blood cells. Shi et al. (2014) also found potent antioxidant activity of ESM hydrolysate or peptides in human intestinal epithelium for gut healing. In another study, it was demonstrated that both processed ESM powder and its derivatives have immunomodulation and anti-inflammatory effects on immune cells, suppressing the activity of nuclear factor-κB (NF-κB), suggesting potential use as wound dressing material (Vuong et al., 2017). Recently, Savic Gajic et al.(2021)also encapsulated orange peel carotenoids in Ca-alginate beads to protect the antioxidant activity. The alginate encapsulation of natural components improved the anti-oxidative and anti-inflammatory efficiency of the bio-polymeric beads.

### *In vitro* biocompatibility

**FIGURE 10:**
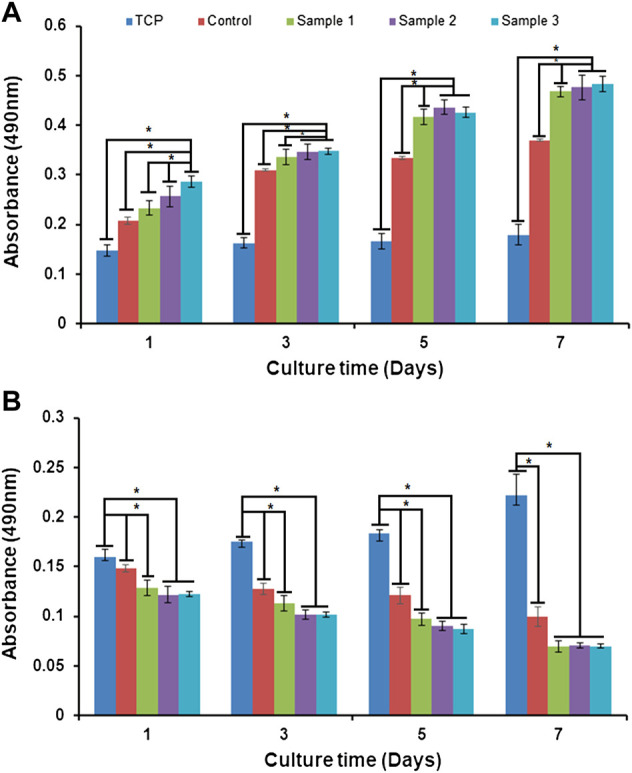
*In vitro* cell biocompatibility *via* MTT assay of **(A)** fibroblast cells and **(B)** carcinoma cells cultured in tissue culture plates (TCP), and with control beads or ESMP-modified sample beads. Here, * indicates the significant difference between samples (*p* < 0.05).

Biopolymers, alginate, and plant–animal products are naturally biocompatible and therefore serve as non-toxic sources of components widely used in biomedical applications. Alginate is a dressing material for wound healing, encapsulation, and controlled release of biomolecules or drugs in the biological system (Dave, 2019). Additionally, the phenolic components of *Aloe vera* significantly stimulate cell proliferation and migration of skin cells to enhance wound healing (Teplicki et al., 2018). In our study, the MTT assay of cells cultured on the bio-polymeric beads showed cell viability, proliferation, and growth over time (Figure 10). The bio-polymeric matrix showed significant increases in the absorbance of samples cultured with fibroblast cells compared to carcinoma cells. The absorbance of the sample represented the cell growth, with constant increase after incorporation of ESMP components in all modified beads, which indicated biocompatibility. However, in the case of carcinoma cells cultured on beads, cell number and growth gradually decreased over time in all samples, representing toxic effects of their anti-inflammatory and anti-oxidative properties.

In another study, a bio-synthetic, biomimetic, nanofibrous scaffold containing soluble ESM and *Aloe vera* for repairing cutaneous tissue showed improved cell proliferation over time compared to the control PVA-silk fibroin film (Mohammadzadeh et al., 2019). Recently, fabrication and evaluation of the non-toxic effects of alginate film modified with *Aloe vera* gel and cellulose nanocrystals for wound dressing application were studied (Thomas et al., 2020). The natural 3D porous polymeric matrix enriched with natural anti-oxidative, anti-inflammatory, and growth-promoting molecules resulted in good biocompatibility and anti-cancerous properties of the bio-polymeric beads. The results showed higher cell viability and proliferation with the natural bio-polymeric beads compared to the TCP, which was used as a control.

## Conclusion

In this study, the bio-polymeric beads of alginate, orange peel, *Aloe vera*, and ESMP components showed good physiological and *in vitro* biological properties. We know that we need healthy cells for a healthy tissue or organ, and cell ageing is the leading cause of prolonged healing with non-functional tissue regeneration. Today, ageing, from the cellular to the individual level, is a significant problem due to unhealthy lifestyle. There are many anti-ageing products available, but at high cost. Thus, our study to cure or slow down the cell ageing process by focusing on natural by-products showed significant outcomes, such as good hydrophilicity and antibacterial, anti-inflammatory, and anti-oxidative properties that result in better cell growth. The fabricated beads consist of natural bioactive components. They are evaluated as biocompatible products *in vitro* without any significant toxic effects on regular cell lines and *vice versa* for the cancerous cell line. Thus, the fabricated natural polymeric bead components (ESMP, orange peel, *Aloe vera*) enhance their therapeutic index for slowing the ageing process and are potentially applicable as regenerative medicine for bio-functional tissue. We conclude that *in vivo* or pre-clinical trials must be performed to prove the commercial applicability of the beads as consumer-friendly products that can be used as cosmetic or therapeutic products in the same manner as current commercially available dermatological gels, creams, face wash, scrubs, and cleansing agents.

## Data Availability

The raw data supporting the conclusion of this article will be made available by the authors, without undue reservation.
