# Corrigendum: Fabrication and evaluation of herbal beads to slow cell ageing

**DOI:** 10.3389/fbioe.2023.1313671

**Published:** 2023-10-19

**Authors:** Archna Dhasmana, Sumira Malik, Amit Kumar Sharma, Anuj Ranjan, Abhishek Chauhan, Steve Harakeh, Rajaa M. Al-Raddadi, Majed N. Almashjary, Waleed Mohammed S. Bawazir, Shafiul Haque

**Affiliations:** ^1^ Himalayan School of Biosciences, Swami Rama Himalayan University, Jolly Grant, Dehradun, Uttarakhand, India; ^2^ Amity Institute of Biotechnology, Amity University Jharkhand, Ranchi, Jharkhand, India; ^3^ Department of Biotechnology, Dr KNMIPER, Modinagar, Uttar Pradesh, India; ^4^ Academy of Biology and Biotechnology, Southern Federal University, Rostov-on-Don, Russia; ^5^ Amity Institute of Environmental Toxicology, Safety and Management, Amity University, Noida, India; ^6^ King Fahd Medical Research Centre, King Abdulaziz University, Jeddah, Saudi Arabia; ^7^ Department of Community Medicine, Faculty of Medicine, King Abdulaziz University, Jeddah, Saudi Arabia; ^8^ Department of Medical Laboratory Sciences, Faculty of Applied Medical Sciences, King Abdulaziz University, Jeddah, Saudi Arabia; ^9^ Hematology Research Unit, King Fahd Medical Research Centre, King Abdulaziz University, Jeddah, Saudi Arabia; ^10^ Animal House Unit, King Fahd Medical Research Centre, King Abdulaziz University, Jeddah, Saudi Arabia; ^11^ Medical Laboratory Technology Department, Faculty of Applied Medical Sciences, King Abdulaziz University, Jeddah, Saudi Arabia; ^12^ Research and Scientific Studies Unit, College of Nursing and Allied Health Sciences, Jazan University, Jazan, Saudi Arabia

**Keywords:** herbal, quercetin, drug, graft, biocompatibility, herbal extracts

In the published article, there was an error in the Funding statement. [grant no. (IFPIP:1866-141-143], one missing digit (i.e., one more 4 to be added which refers the Hijri year). The correct statement appears below:

Acknowledgments

“This research work was funded by the Institutional Fund projects under grant no. (IFPIP:1866-141-1443). The authors gratefully acknowledge technical and financial support provided by the Ministry of Education and King Abdulaziz University (KAU), Deanship of Scientific Research (DSR), Jeddah, Saudi Arabia.”

The authors apologize for this error and state that this does not change the scientific conclusions of the article in any way. The original article has been updated.

